# An integrated epigenome and transcriptome analysis identifies PAX2 as a master regulator of drug resistance in high grade pancreatic ductal adenocarcinoma

**DOI:** 10.1371/journal.pone.0223554

**Published:** 2019-10-17

**Authors:** Imlimaong Aier, Rahul Semwal, Aiindrila Dhara, Nirmalya Sen, Pritish Kumar Varadwaj

**Affiliations:** 1 Department of Bioinformatics & Applied Sciences, Indian Institute of Information Technology—Allahabad, Uttar Pradesh, India; 2 Department of Information Technology, Indian Institute of Information Technology—Allahabad, Uttar Pradesh, India; 3 Cancer Research Program, Rajiv Gandhi Centre for Biotechnology, Trivandrum, Kerala, India; 4 S.N.Bose Innovation Centre, University Of Kalyani, Nadia, West Bengal, India; University of Texas Health Science Center at San Antonio, UNITED STATES

## Abstract

Pancreatic ductal adenocarcinoma (PDAC) is notoriously difficult to treat due to its aggressive, ever resilient nature. A major drawback lies in its tumor grade; a phenomenon observed across various carcinomas, where highly differentiated and undifferentiated tumor grades, termed as low and high grade respectively, are found in the same tumor. One eminent problem due to such heterogeneity is drug resistance in PDAC. This has been implicated to ABC transporter family of proteins that are upregulated in PDAC patients. However, the regulation of these transporters with respect to tumor grade in PDAC is not well understood. To combat these issues, a study was designed to identify novel genes that might regulate drug resistance phenotype and be used as targets. By integrating epigenome with transcriptome data, several genes were identified based around high grade PDAC. Further analysis indicated oncogenic PAX2 transcription factor as a novel regulator of drug resistance in high grade PDAC cell lines. It was observed that silencing of PAX2 resulted in increased susceptibility of high grade PDAC cells to various chemotherapeutic drugs. Mechanistically, the study showed that PAX2 protein can bind and alter transcriptionally; expression of many ABC transporter genes in high grade PDAC cell lines. Overall, the study indicated that PAX2 significantly upregulated ABC family of genes resulting in drug resistance and poor survival in PDAC.

## Introduction

Pancreatic cancer is the fourth leading cause of cancer related death in several countries and will surpass breast, prostate cancer to become the second leading cause of cancer related death in the near future [1], [2], [3]. With no apparent symptoms in its early stages, pancreatic cancer grows aggressively, infiltrating adjacent tissues and promoting metastasis along with resistance to chemo and radiotherapy [4], [5]. Most of the patients are present with metastatic disease or local infiltration at the time of initial diagnosis, and only 15–20% of the patients are candidates for surgical resection. Out of the several types of pancreatic cancer, pancreatic ductal adenocarcinoma (PDAC) is the most common (85%) [6].

Clinically, histological alterations indicating more differentiated PDACs are termed as low grade, while the undifferentiated tumors are termed as high grade PDAC [7], [8]. The clinical aspects that might help PDACs develop drug resistance includes tumor heterogeneity, epithelial mesenchymal transition, and upregulated drug transporters [9], [10], [11]. Many ABC family of transporters, like *ABCC1*, *ABCC2*, *ABCC3*, and *ABCC5* have been shown to be upregulated in pancreatic cancer patients [12], [13], [14], [15], [16], [17], [18]. Several studies have established the relationship between ABC family transporter proteins and drug resistance in pancreatic cancer. *ABCC5* expression, for instance, was found to be significantly correlated with cellular sensitivity to 5-fluorouracil (5-FU) [14] and gemcitabine [18], where acquired resistance to 5-FU was associated with an increase in *ABCC5* expression [14]. Further, it was reported that expression levels of *ABCC3* and *ABCC5* changed during tumor development, and the expression of *ABCC3* was significantly correlated with high tumor grade. It was also observed that *ABCC3* expression was linked to survival in patients, where patients with lower expression of the gene had longer survival period [12]. Upregulation of *ABCC2* and *ABCC1* is often expressed in a PDAC cohort [19]. *ABCC2* is correlated with negative prognosis in several forms of carcinomas, while *ABCC1* expression reportedly increased when exposed to high doses of gemcitabine in PDAC [20]. However, a distinct regulation of these transporters with respect to tumor grade is not well understood.

To understand the underlying mechanism of PDAC behavior due to its heterogeneity, an integrative study was conducted to identify enriched regions within the genome marked by histone methylation regulators, and to correctly assign the upregulation and downregulation of important genes present within these regions. In addition, network study to understand the underlying biological process and pathway of significantly enriched genes present in the high grade cell lines were conducted. Oncogenic transcription factor PAX2 (belonging to the conserved DNA-binding paired box domain family) showed strong physical network in PDAC cell lines belonging to high grade tumors. Additionally, it was found that PAX2 silencing in pancreatic cancer cells increases susceptibility towards known clinical agents. Our study reveals that PAX2 transcriptionally regulates the expression of several drug transporter family members like *ABCC1*, *ABCC2*, *ABCC3*, and *ABCC5* by binding to the promoters of these drug resistance genes. Overall, this study establishes data integration and mining methods to identify and mechanistically validate genes regulating high grade PDAC related oncogenesis.

## Materials and methods

### Data retrieval, quality check and sequence alignment

The data for the analysis were obtained from NCBI GEO database, deposited by Nicoli P et al [8]. Details about the cell lines used for the study are given below (Table 1):

**Table 1:** ChIP-Seq and RNA-Seq datasets with corresponding ID.

	Cell-line	GEO ID			
Low grade PDAC		ChIP-Seq		RNA-Seq	
		H3K4Me1	H3K4Me3	Replicate 1	Replicate 2
	Control	GSM1574271			
	CAPAN-2	GSM1574243	GSM1574258	GSM1574299	GSM1574300
	CFPAC-1	GSM1574245	GSM1574259	GSM1574301	GSM1574302
	High grade PDAC	Control	GSM1574272			
		MIA PaCa-2	GSM1574250	GSM1574261	GSM1574305	GSM1574306
PANC-1		GSM1574252	GSM1574262	GSM1574307	GSM1574308

Quality check on the raw data files were done using FastQC (http://www.bioinformatics.babraham.ac.uk/projects/fastqc), a tool for assessing the quality of NGS data. For RNA-Seq data, HISAT2 [21] was used for alignment of sequences due to its speed and sensitivity. Forward and reverse strands of each replicates were aligned using built-in genome for reference (hg19). The mean distance between pairs was set to 200bp for each sample.

### Read count and Expression analysis

FeatureCounts [22], a highly optimized read counting tool was used for the study because of its speed and reliability. Aligned RNA-Seq reads in the form of Binary Alignment Map (BAM) was provided as input, along with a list of genomic features in Gene Transfer Format (GTF) for human reference genome Human GRCh37 (hg19). The procedure was carried out for all the replicates. DeSeq2 [23], a package in R for differential gene expression, was used for this study. The dataset were divided into low grade group, consisting of CAPAN-2 and CFPAC-1 replicates, and high grade group, consisting of MIA PaCa-2 and PANC-1 replicates. Differential gene expression analysis was performed by considering the low grade group as control, and the high grade ones as treatment.

ChIP-Seq data for PDAC were aligned using Bowtie2 [24], a fast memory efficient tool for large sequence alignment. All reads were single-end, and were mapped using built-in genome index with hg19 as the reference genome. The computational analysis of ChIP-Seq experiment started with fastq files containing an associated per-nucleotide quality score Q that is an estimation of the -10*log (p), where p is the probability that the corresponding base call is incorrect. The pipeline involves quality assessment by plotting base qualities and frequencies per sequencing cycle, alignment to the genome, duplicate filtering of alignments, removal of low complexity regions and transformation of the aligned data to coverage vectors.

### Peak calling and peak correlation

MACS2 [25] is a program that is widely considered the best tool for ChIP peak identification. It makes use of a parametric model based on a local Poisson distribution parameterized from the control data. The filtered and aligned dataset obtained from the previous step was then provided as input for peak calling and identification. The enriched regions for H3K4Me1 and H3K4Me3 were discovered using default settings with BW = 300, along with the broad peaks option. The comparison for each sample was made with its corresponding input data.

Correlation between the peaks of all datasets were compared using deep sequencing tools [26] present in the Galaxy platform [27] for visualization of deeply sequenced data. BAM files generated by Bowtie2 were processed using the module bamCoverage to reduce the size of data. This resultant data was saved in bigwig format. The data was then compared against each other based on their difference using the module bigwigCompare. Correlation plot of all the datasets were then computed using Spearman correlation coefficients. EaSeq [28], a tool for ChIP-Seq data analysis and visualization was used for annotation of genes to peak regions, and the peak intensity of low grade and high grade PDAC peaks were compared. Visualization of peak regions around genes were carried out using UCSC browser [29].

### Integrative analysis of ChIP and RNA-Seq data using BETA, Gene Ontology and network analysis

Binding and Expression Target Analysis (BETA) [30] is an integrative tool for the analysis of transcription factors and chromatin regulator binding sites from ChIP-Seq by correlating with differentially expressed genes from RNA-Seq. The main purpose of this tool is to determine the activating and repressing function of each gene, which is detected using a nonparametric statistical test.

For this study, BETA was used for the integration of ChIP with RNA-Seq. Peak files of each cell line were correlated with their corresponding differentially expressed gene data according to their methylation state. The operation was carried out using hg19 as the reference genome, and by setting the Benjamini-Hochberg false discovery rate (FDR) for differentially expressed genes at ≤ 0.05. The result from this analysis consisted of a list of upregulated and down regulated genes along with their ranks based on the regulatory potential of factor binding and differential expression upon factor binding.

Enrichment analysis of high-throughput data was calculated inclusive of parameters like Chi-square, Fisher's exact test, Binomial probability and Hypergeometric distribution [31]. The genes identified from high-throughput screening are then annotated with Gene Ontology (GO) [32] terms. Cytoscape [33], an open source tool for visualization and analysis of complex networks, was used for the analysis of expressed gene data network. Cytoscape gives the ability to integrate arbitrary data on the graph, while serving as a platform for its visual representation. Moreover, the interface has the means to implement external methods in the form of plug-ins. Biological Networks Gene Ontology tool (BinGO) [34], a plugin for ontology analysis in Cytoscape, was used for ontological analysis of biological processes, and the connections between selected genes were studied using GeneMANIA [35], a tool for generating hypothesis on gene function and analysis of gene sets. For BinGO, overrepresented genes were specified for the visualization, which were selected using a hypergeometric test, with an FDR of 0.05. GO biological process was selected as the preferred ontology process for *Homo sapiens*, exclusively. GeneMANIA parameters were specifically restricted to genetic interactions, physical interactions, and shared pathways, to further isolate and study relevant connections between selected genes. All weightage provided were set to automatic. Comparison between the upregulated and downregulated genes of high grade data, and analysis of key genes were visualised in the form of a Venn diagram using the tool FunRich [36], an enrichment tool which provides graphical output for data visualization. FunRich was also used for assigning ontology process to a few selected genes from UniProt database [37] for an FDR of ≤ 0.05. Pathway analysis was conducted using ClueGo [38] by selecting KEGG pathways for the desired ontology. Genes that occur below a threshold of p-value ≤ 0.05 were investigated at global level network specificity. Pathways relevant to cancer were investigated using Pathview [39], with the help of individual cell line gene expression data from DEGUST (http://degust.erc.monash.edu/) using edgeR [40]. HGPEC [41], a plugin for Cytoscape which uses a random walk with restart on heterogeneous data (RWRH) algorithm, was used to predict the disease-gene association on heterogeneous network. In this method, a list of known genes from given diseases are used as training data. A complete different set of genes, which do not appear on the training set of genes, serve as the candidate gene set. The RWRH algorithm then uses the training data to identify and classify all candidate genes and disease in the heterogeneous network.

### Cell culture, reagents and transfection

MIA PaCa-2 (MIA PaCa-2 ATCC® CRL-1420^™^) cells and PANC-1 (PANC-1 ATCC® CRL-1469^™^) cells were provided by Dr. Hari Kumar KB (RGCB, India). Both cells were cultured in DMEM medium, (Thermo Fisher Scientific, Waltham, MA), supplemented with 10% fetal bovine serum (FBS), at 37°C, 5% CO2. The identity of cell lines were confirmed by STR (Short Tandem Repeats) profiling using standard primer sets for core human loci (https://strbase.nist.gov/coreSTRs.htm) and the cells were determined to be mycoplasma free using MycoAlert Mycoplasma detection kit from Lonza (NJ, USA). For RNAi studies, previously validated PAX2 siRNA (5’CATCAGAGCACATCAAATC3’, [42]) (Integrated DNA Technologies, Inc., USA) was used and cells were transfected using 20 nM siRNA complexed with RNAi‐Max (Thermo Fisher Scientific).

The siCell Death, siRNA used as Positive cell death phenotype control, from qiagen, USA. This commercially available siRNA known as AllStars Hs Cell Death siRNA (Catalogue Number SI04381048). Camtothecin and 5’Flurouracil were purchased from Tocris biosciences and dissolved according to instructions. Gemcitabine was purchased from Santa Cruz biotechnology and dissolved as per instructions.

### mRNA analysis

For real‐time PCR analysis, total RNA from cells was extracted using RNA extraction kit (Thermo Fisher Scientific, MA), and cDNA was synthesized using the iScript cDNA synthesis kit (Bio‐Rad, Hercules, CA) following the manufacturer's protocol. For normalization 18S ribosomal RNA was used as a reference. The following primer sets were used for SYBR green based qPCR: PAX2: Forward primer 5′‐ CAACGGTGAGAAGAGGAAACGAG ‐3′, Reverse Primer 5′- TAATGCTGCTGGGTGAAGGTGTC-3’ [43], 18sRNA: Forward 5’- GTAACCCGTTGAACCCCATT-3’, Reverse 5’- CCATCCAATCGGTAGTAGCG 3’, ABCC2/MRP2: Forward 5’- ACGGGCACATCACCATCAAG -3’, Reverse 5’- CTCCAGGCAGCATTTCCAAG -3’ [44], ABCC3/MRP3: Forward 5’- CGCCTGTTTTTCTGGTGGTT -3’, Reverse 5’- TTGTGTCGTGCCGTCTGCTT -3’ [44], ABCC1/MRP1: Forward 5’- ACCCTAATCCCTGCCCAGAG -3’, Reverse 5’- CGCATTCCTTCTTCCAGTTC -3’ [44], ABCC5/MRP5: Forward 5’- CCAAGCTGACCCCCAAAATGAAAAA -3’, Reverse 5’- TGGATGTGCTTGCCTTCTTCCTCTTC -3’ [44], TM4SF1: Forward 5’- AAGGGGGAGAAAACCTAGCA -3’, Reverse 5’- CCAGCCCAATGAAGACAAAT -3’ [45].

### Chromatin Immunoprecipitation assays

ConTra V3 [46] was used for the identification of TFBS across the promoter regions of ABC transporter family of genes, which was further verified using the GTRD [47]. This process was carried out to find conserved regions of PAX2 protein, and to extract these features for the designing of primers. Chromatin immunoprecipitations were carried out using a kit following the manufacturer’s protocol (Millipore). Briefly, 1x10^7^ cells were fixed with formaldehyde and lysed with SDS Lysis Buffer. The cell lysates were then sonicated to shear the DNA to lengths between 0.2-1kb. The samples were precleared with Protein agarose slurry. Control normal Rabbit IgG (Catalogue No#2729, Cell Signalling Technology, USA) or Anti-PAX2 Rabbit (Catalogue No# ab23799, AbCam Plc, UK) antibody was added (concentration 10μg/ml) and incubated overnight at 4°C followed by incubation with fresh Protein agarose slurry for 2 hours. Precipitated chromatin complexes were removed from the beads through 30-min incubation with 500 μL of elution buffer. Finally, the protein–DNA cross-links were reversed by incubation at 65°C for 4 hours and immunoprecipitated DNA was analyzed by qPCR. Primers used for this analysis are given below:

ABCC2: 5’GGACCACTCTGCTTATCTTGGA3’; 5’GGTGTGCTGCAGTGTAAATCA 3’

ABCC5: 5’ GATAATCAGCTAAGCTAGGGAAC3’; 5’CCATAAAGAACAGCGGCTCAC3’

ABCC1: 5’GGTGTCTCTCTGCTTCTGT3’; 5’CTTTCTACAATCTAAGCCCC3’

ABCC3: 5’ ATGAAGGCAGAGCTGGGATA3’; 5’ GTGCGATCATAGCTCACAGTA3’

TM4SF1: 5’ CCAGGCTGGTCTTGAACTCAT3’; 5’CGAAAGAAACTTAAGTGTGATG3’

### Cell viability and caspase assay

Caspase activation and cell viability was measured using the CaspaseGlo 3/7 and the CellTiter‐Glo® Luminescent assay systems respectively (Promega) as per manufacturer's instruction using at least three biological replicas of each treatment. Spark10M luminometer from Tekan was used for luminescence measurement.

### Statistical analysis

Statistically significant differentially expressed genes and BETA analysis were identified using Benjamini-Hochberg false discovery rate. Enriched ChIP-Seq peaks were identified using empirical false discovery rate. For gene ontology studies, hypergeometric test and Benjamini-Hochberg false discovery rate were performed. Statistical analysis was performed for all wet lab experiments. Experiments were performed as biological triplicates. Dunnette’s multiple comparison test, Student t-test were performed for cell survival data. For real time PCR data, SEM ±3 of biological triplicates were considered. All data were found to be statistically significant.

## Results and discussion

### Analysis of PDAC dataset

The quality of dataset used for analysis of PDACs were verified using FastQC and the mean quality scores of all cell lines indicated that the data lies in the good quality call region with minimal chances of error. The total number of expressed genes obtained were 20,795, out of which the number of genes classified as differentially expressed were 1044 over-expressed and 2092 under-expressed genes based on an FDR of 0.05 (5%) and fold enrichment > 2. Fig for correlation between the samples, and the volcano plot for differentially expressed genes were provided (S1 Fig), while a comprehensive list of all up/downregulated genes can be found in S1 Table. Genes below this particular threshold were discarded from further use.

**Fig 1:**
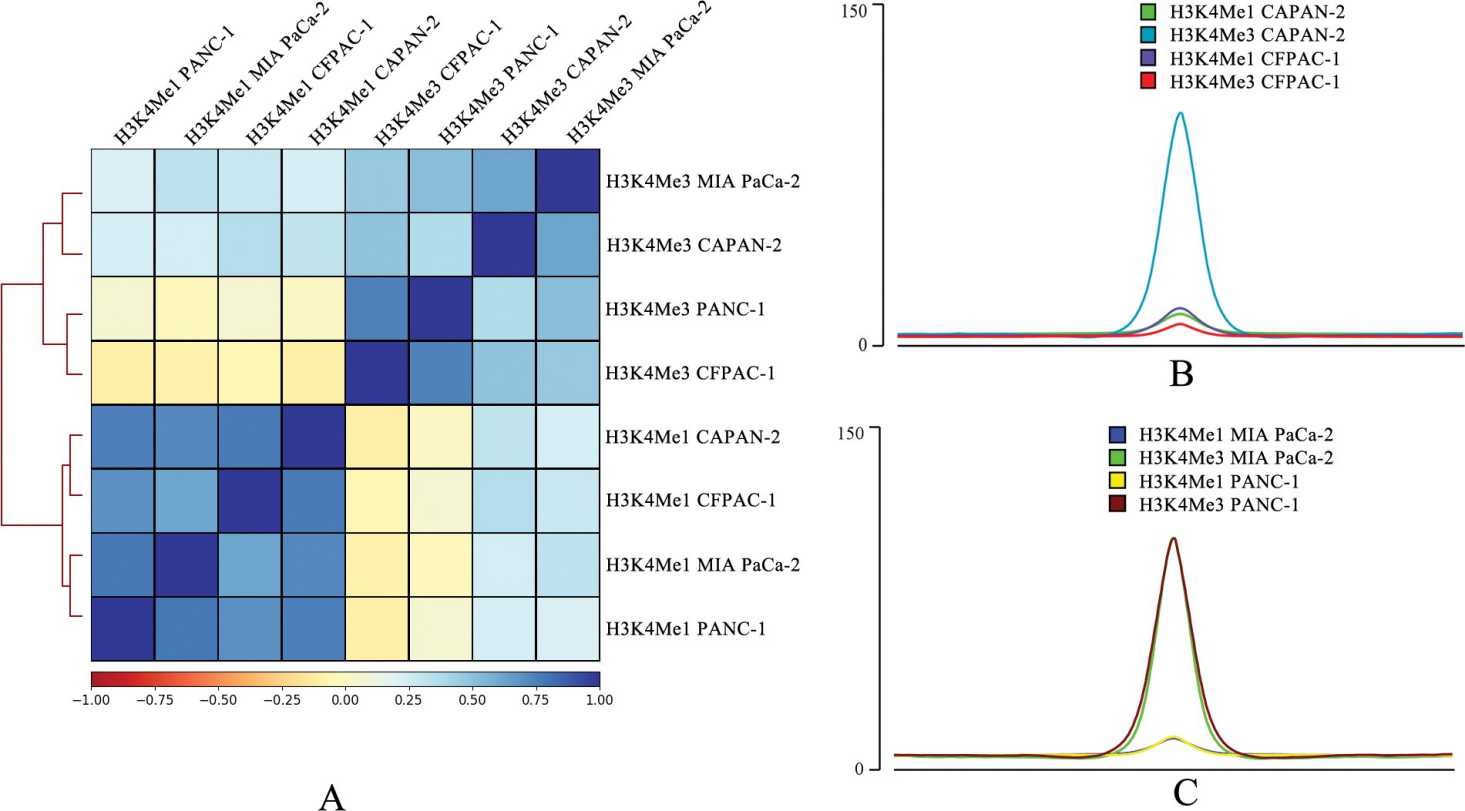
Correlation plot for high grade PDAC ChIP-Seq dataset and peak signal intensity. (A) Spearman correlation plot for low and high grade PDAC ChIP-Seq dataset. Correlation comparison of peak signal intensity for (B) Low grade PDAC cell lines and (C) High grade PDAC cell lines.

PDAC dataset was analyzed for activating methylation signatures (Monomethylation; H3K4Me1 vs. Trimethylation; H3K4Me3) in MIA PaCa-2 and PANC-1 cells belonging to the high grade PDAC cell lines (Fig 1). Low grade PDAC cell lines represented by CFPAC-1 and CAPAN-2 were also used as controls during data analysis.

During signal intensity comparison between methylated peaks (Fig 1A), the monomethylated peaks for low grade lines (CAPAN-2 and CFPAC-1), displayed a deep shade of blue, corresponding to greater correlation. In the case of trimethylated peaks for low grade samples, correlation between CAPAN-2 and CFPAC-1 became distant, with CAPAN-2 H3K4Me3 peaks clustering more towards trimethylated peaks representing High grade cell lines (Fig 1B). Monomethylated and trimethylated high grade samples, MIA PaCa-2 and PANC-1, on the other hand, displayed good correlation, indicating close association between the cell lines. High grade PDAC cell line (MIA PaCa-2 and PANC-1) peak frequencies were on par for H3K4Me3, with a high intensity. Although H3K4Me1 peak intensities for MIA PaCa-2 and PANC-1 were on the lower end, very similar peak profiles were observed (Fig 1C). Overall, peak intensity for high grade PDAC were closely matched for all cell lines. On the other hand, low grade PDAC peak intensity for each cell line was varied (Fig 1B). The results from methylation pattern comparison indicated that high grade tumors are more likely to show similar gene expression for methylated promoter regions.

**Fig 2:**
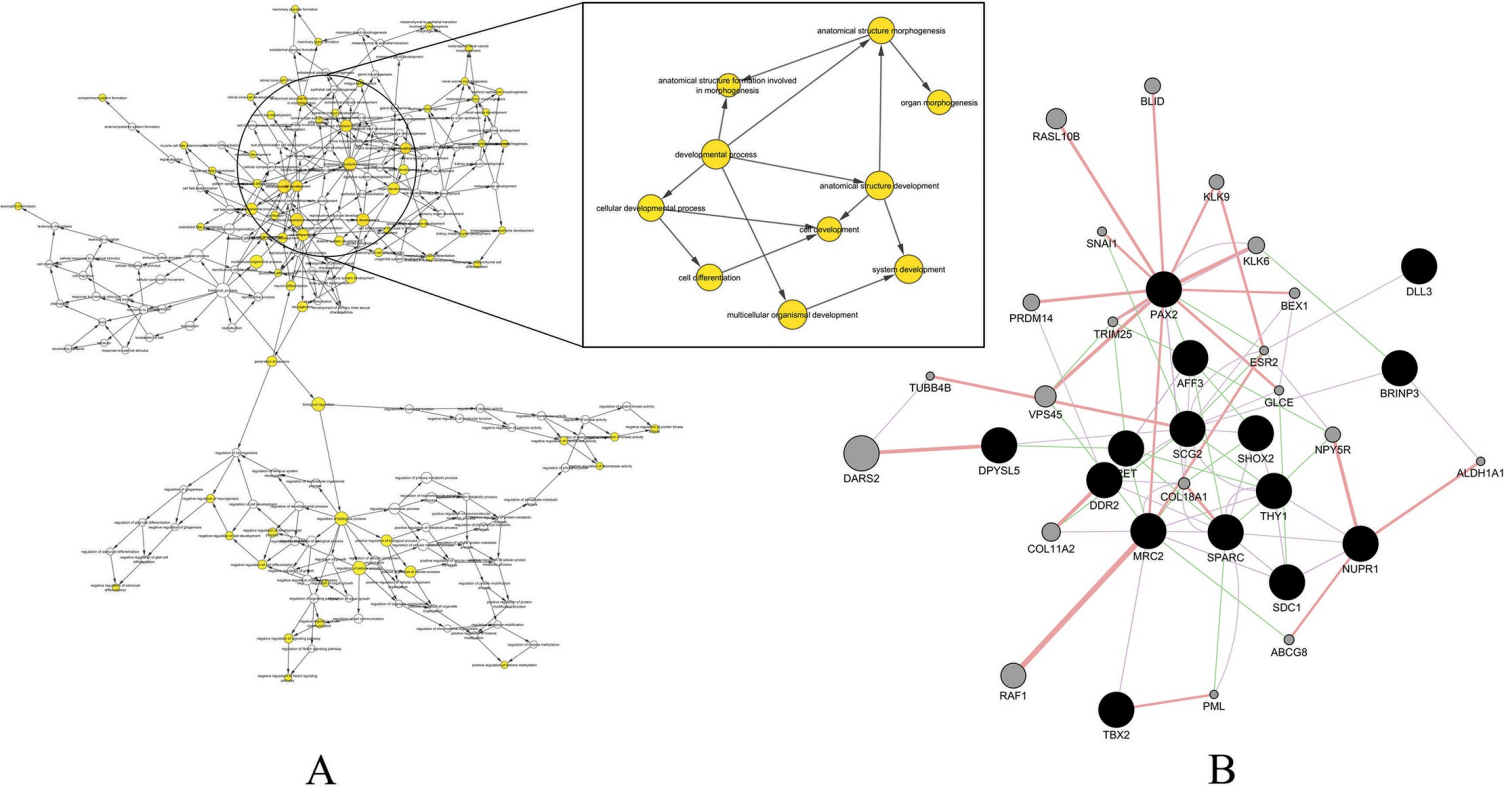
Analysis of high grade PDAC dataset for differential expressed genes using Cytoscape. (A) Representative network cluster for biological process that are actively involved in cell differentiation and structural development were identified using BinGO. Nodes represented in white were not significantly expressed, while a deeper shade of orange correlated with processes predicted with confidence. The inset represents a cluster of biological processes with p-value ≤ 0.05. These clusters were involved in cell differentiation and structural formation. (B) Gene interaction network obtained using GeneMANIA was conducted on selected genes identified from important biological processes. Black nodes represent the selected genes, while grey nodes were automatically drawn by the tool based on their connectivity to the highlighted ones. All edges in the network were derived from prior knowledge.

To analyze the differentially expressed genes associated with altered methylation in high grade lines, BETA Plus analysis was performed on high grade cell lines by integrating ChIP-Seq data with RNA-Seq data, thus providing a list of upregulated and downregulated genes. Activation and repression function of histone methylation were predicted from PDAC cell lines, and upregulated/downregulated genes were visualized in the form of Venn diagrams (S2 and S3 Figs). Cytoscape and BinGO tools identified a large interconnected cluster, displaying affinity towards cellular differentiation and structure development (Fig 2A), with optimal FDR. This interconnected network was further isolated and the participating gene components, with a strong relation to cancer, were verified through text mining. This group of genes were then analyzed using GeneMANIA to decipher the interconnection between them. The criteria for the interconnection were specifically assigned for co-expression, and physical and genetic interactions. From the generated network (Fig 2B), it was found that *PAX2* gene had the highest number of physical interactions, thus making it an important target. Other genes that were highlighted in the network like *DDR2*, *DPYSL5*, *SPARC*, and *FAM5C* were also evaluated to understand their contribution in the growth and resistance of PDAC. Additional ontology analysis for each of the genes mentioned above were conducted to disseminate their individual roles (S4 Fig).

**Fig 3:**
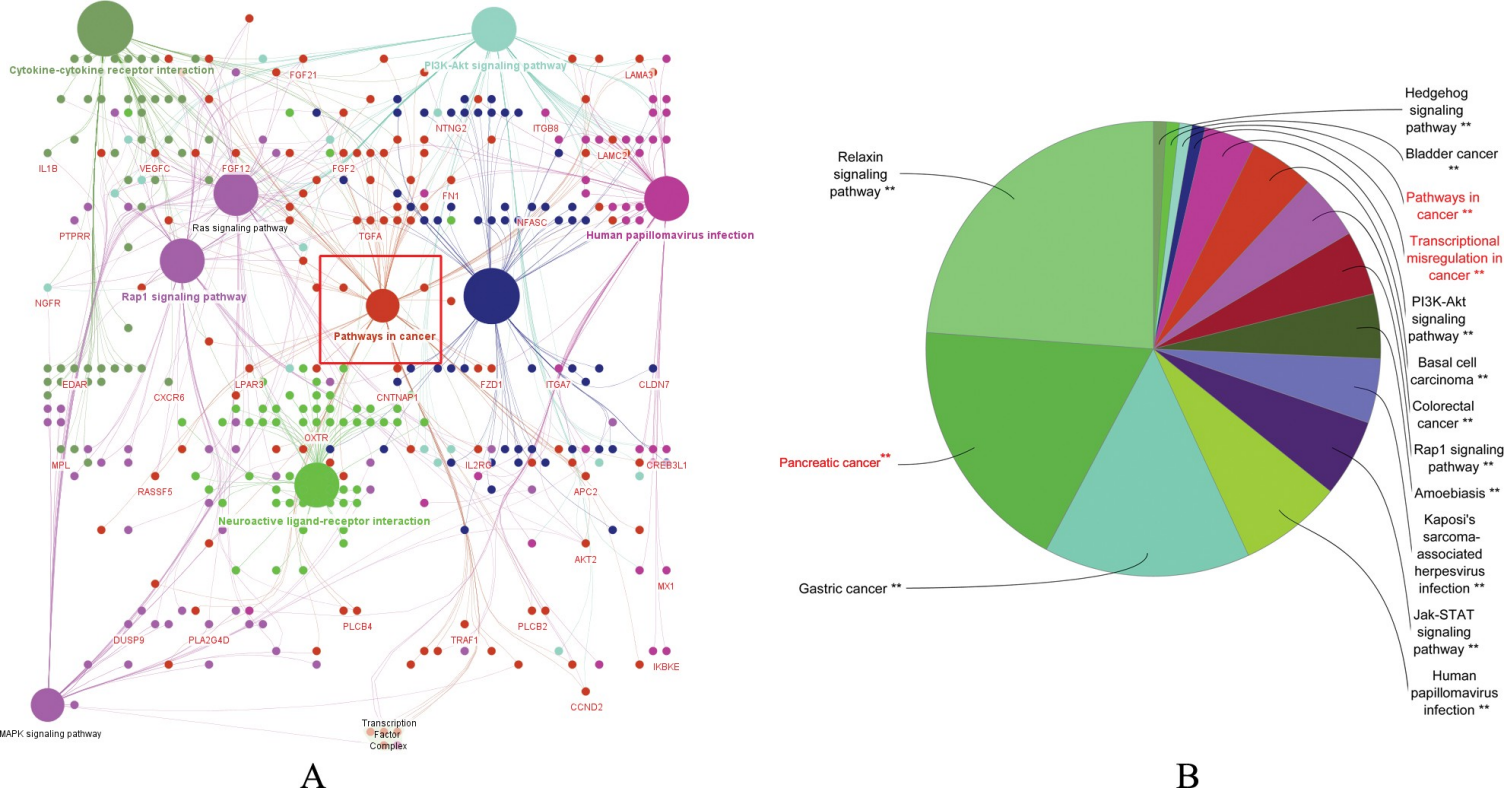
Pathway analysis using ClueGO indicated that several identified genes were interconnected to cancer related pathways. **(**A) Representation of KEGG pathways enriched in PDAC gene set (Bonferroni step-down corrected p ≤ 0.05). Pathways in cancer (highlighted in a red box) was found to be interconnected to other over-represented terms (B) Pie-chart of pathways expressed in PDAC for gene set extracted from pathways in cancer, with essential pathways marked in red.

The network of significant pathways predicted by ClueGo was depicted in Fig 3A, where pathways in cancer was highlighted in a red box. It was observed that a majority of the genes were involved in this pathway, and were interconnected with several other essential processes. To further investigate this set of genes, the tool was rerun, but this time at medium level network specificity for a more detailed insight into the pathways involved in cancer. As expected, the genes that partake in pathways in cancer were seen in pancreatic cancer, as well as transcriptional misregulation in cancer (Fig 3B). To understand the contribution of each gene, the fold enrichment for individual cell lines were obtained using DEGUST. Gene expression data from DEGUST output was divided into groups: control (low grade) and sample (high grade), which was then used to generate KEGG pathways for pathways in cancer, transcriptional misregulation in cancer and pancreatic cancer using Pathview (S5, S6, and S7 Figs).

**Fig 4:**
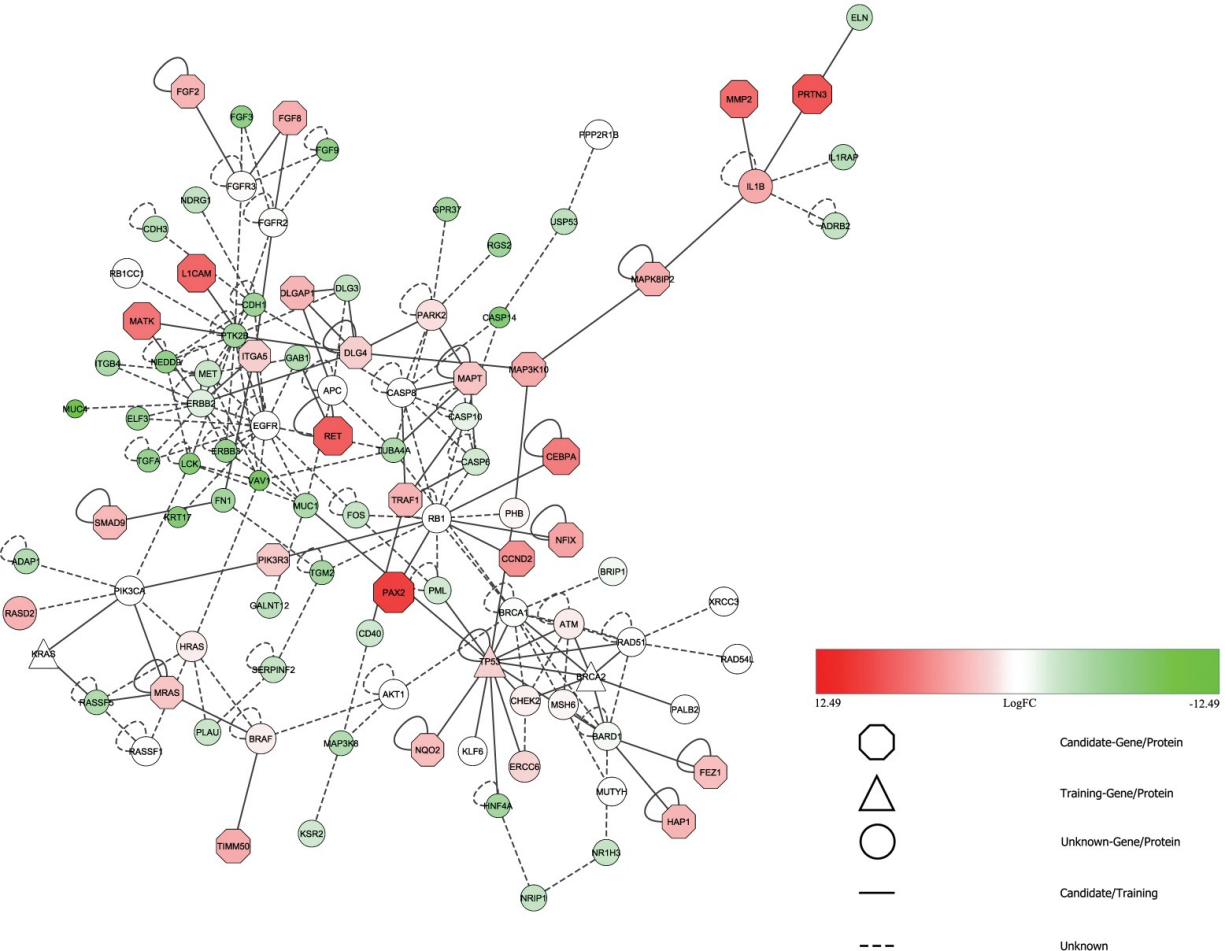
Predicted gene topological relation as obtained from HGPEC.

The evidence of *PAX2* as an essential gene in the regulation of high grade PDAC was further solidified using HGPEC. Initially, genes from disease closely related to pancreatic cancer were selected from DisGeNET database [48] present in HGPEC. This set of genes were used as training dataset. For the candidate gene set (test dataset), the genes identified from the current study were used as input. This provided us with a comprehensive network of genes predicted to be involved in pancreatic cancer (Fig 4).

To biologically validate our findings, we silenced individual gene like *PAX2*, *FAM5C*, *DDR2*, *DPYSL5*, and *SPARC* in high grade PDAC cell line MIA PaCa-2 using RNAi and accessed cell viability to understand critical function of the genes. We found that mRNA levels of all the genes were downregulated upon RNAi but only *PAX2* downregulation showed consequent decrease in cell viability (~37%) in MIA PaCa-2 cells (S8 Fig).

The octagonal nodes were used to represent candidate genes that were highly ranked in pancreatic cancer. *PAX2* is shown to interact with *RB1*, a prominent tumor suppressor gene [49].

### *PAX2* downregulation increases drug susceptibility in pancreatic cells

**Fig 5:**
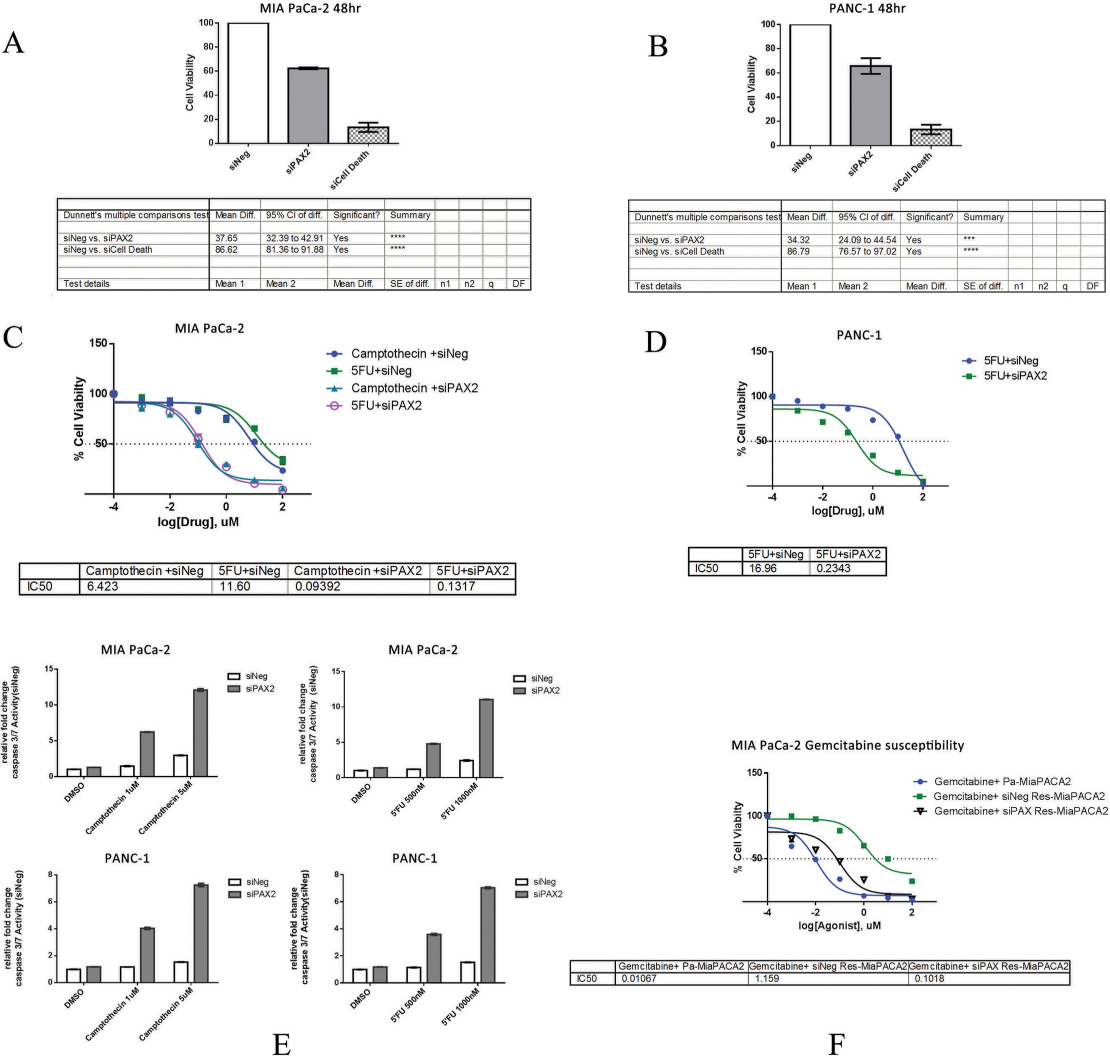
RNAi based silencing of *PAX2* in MIA PaCa-2 and PANC-1 cells led to an increase in drug susceptibility. (A) MIA PaCa-2 and (B) PANC-1 cells were transfected using indicated siRNAs for 48hours. Cell viability assay was performed post 48 hours using luminometer. Percent cell viability was calculated with respect to Negative control siRNA (siNeg). siCell Death, an siRNA used as Positive cell death phenotype control, from qiagen, USA was used as a positive control. Experiments were done independently in triplicates SEM = ± 3. The viability of (C) MIA PaCa-2 and (D) PANC-1 cell lines, previously transfected with siNeg control or siPAX2 siRNA and subsequently (24 hours post transfection) exposed to increasing concentrations of chemotherapeutic drugs camptothecin or 5’Flurouracil for 48 hr (mean ± SEM, n = 3) at each drug concentration normalized to vehicle (V, 0.1% DMSO). (E) Caspase 3/7 activity in MIA PaCa-2 (top panel) or PANC-1 (bottom panel) cell lines, previously transfected with siNeg control or siPAX2 siRNA and subsequently (24 hours post transfection) exposed to indicated concentrations of chemotherapeutic drugs camptothecin or 5’Flurouracil (5’FU) for 48 hours (mean ± SEM, n = 3) at each drug concentration normalized to vehicle (V, 0.1% DMSO). Relative fold change in caspase activity was measured using caspase 3/7 Glo assay using luminometer. (F) The viability of MIA PaCa-2 parental (Pa-MIA PaCa-2) or Gemcitabine resistance MIA PaCa-2 (Res-MIA PaCa-2) cell lines, previously transfected as indicated, with siNeg control or siPAX2 siRNA and subsequently (24 hours post transfection) exposed to increasing concentrations of Gemcitabine for 48 hours (mean ± SEM, n = 3) at each drug concentration normalized to vehicle (V, 0.1% DMSO).

*P*AX2 is known to promote various oncogenic process like metastasis, drug resistance by transcriptionally regulating effector genes in various cancers like Wilms tumor and renal cell carcinoma [50], [51]. Moreover, *PAX2* expression has been linked with EMT transition [52]. However, the role of *PAX2* in high grade PDACs is not known. RNAi based silencing of *PAX2* in high grade PDAC cells (MIA PaCa-2 and PANC-1) resulted in decreased expression of *PAX2* with consequent cell death in MIA PaCa-2 (~37%) and PANC-1 (~34%) cells. Percent cell viability was calculated with respect to Negative control siRNA (siNeg). siCell Death, an siRNA, used as Positive cell death phenotype control (Fig 5A and 5B). Owing to the association of *PAX2* with drug resistance and metastasis in other cancers [53], [54], [55], [56], we were interested to check the effect of *PAX2* silencing with respect to drug treatment in high grade cell lines MIA PaCa-2 and PANC-1 which show more resistance towards drugs like camptothecin, 5’ Flurouracil (5’FU), Gemcitabine as reported previously [11], [57], [58], [13], [59], [60], [61], [62], [63], [64]. *PAX2* was silenced followed by addition of Camptothecin or 5’FU (Flurouracil) at varying concentrations in MIA PaCa-2 cells (Fig 5C). Increased cell death (Camptothecin IC_50_ = 0.094 uM, 5’FU IC_50_ = 0.14uM) was observed in cells that were treated with siPAX2 compared to Negative control siRNA transfected MIA PaCa-2 cells (Camptothecin IC_50_ = 6.42 uM, 5’FU IC_50_ = 11.6 uM). Similar increase in drug sensitivity was observed in PANC-1 treated with 5 FU (5’FU IC_50_ = 16.9uM → 0.24uM) upon *PAX2* silencing (Fig 5D). Caspase3/7 activity of these cells were checked under drug treatment and it was found that *PAX2* silenced cells displayed increased caspase3/7 activity compared to control siRNA transfected cells (Fig 5E). Together, these results suggest that *PAX2* might promote drug resistance phenotype in high grade pancreatic cancers.

Gemcitabine in combination with other agents had shown promising effects in PDACs [63], [65]. However, several factors may cooperatively cause gemcitabine resistance which had been associated with aggressiveness and lethality [66], [67], [68], [69]. We checked for *PAX2* involvement during Gemcitabine resistance in high grade PDAC cells. Increasing dose of gemcitabine (upto 160nM) starting from 10nM treatment was used to generate drug resistant MIA PaCa-2 cells (Res-MIA PaCa-2) as described previously [13]. Interestingly, *PAX2* knockdown in Res-MIA PaCa-2 cells was able to decrease cell viability as compared to control siRNA treated cells, thus reestablishing gemcitabine sensitivity (Fig 5F). This data indicated that *PAX2* plays a critical role in development of various drug resistance in PDACs.

### PAX2 transcriptionally regulates ABC family of transporters

**Fig 6:**
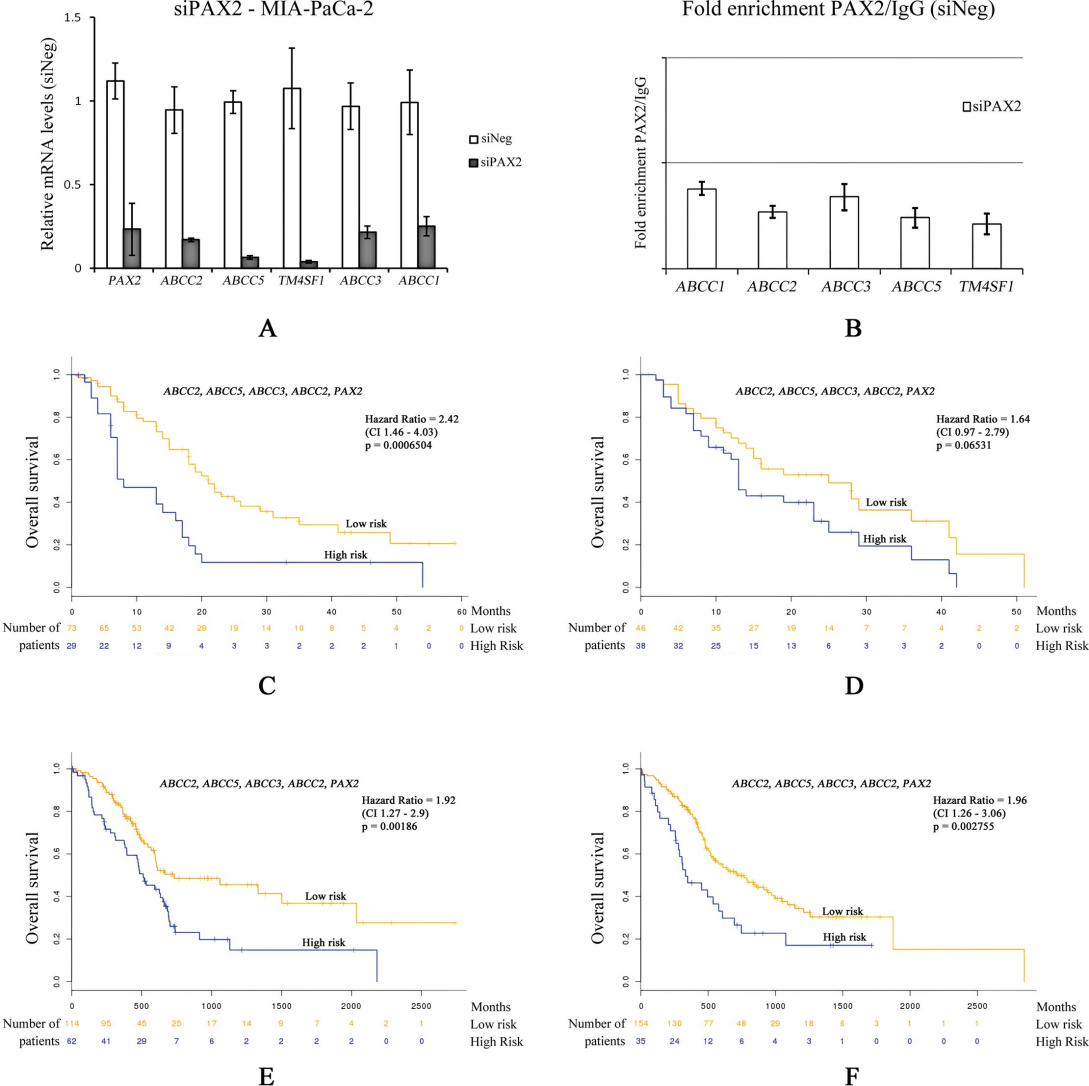
Effect of PAX2 on the transcriptional regulation of *ABC* family of transporters and survival analysis. (A) MIA PaCa-2 cells were transfected with siRNAs against *PAX2*. Relative mRNA levels were analysed with respect to relative mRNA levels (siNEG) 48hours post transfection. Normalization was performed with 18sRNA levels. Experiments were done independently in triplicates SEM = ± 3. (B) MIA PaCa-2 cells were transfected with siNeg or siPAX2 siRNAs. ChIP assay was then performed with *PAX2* antibody or control IgG antibody. The fold enrichment of coprecipitating DNA was determined by qPCR for the indicated promoters. Error bars are means ± SD of three independent experiments with triplicate samples. Survival analysis was performed using Surv Express software [72]. Patient datasets used (C) [73], (D) [74], [75], (E) TCGA pancreatic adenocarcinoma, (F) ICGC portal, for the multivariant analysis of indicated genes *ABCC2*, *ABCC5*, *ABCC3*, *ABCC1* and *PAX2*. High (blue) and low (yellow) represents gene expression.

Various ABC family of transporters are known for promoting drug resistance in pancreatic cancer patients [70], [71]. However, the transcriptional regulation of these transporters in PDACs is not well understood. The expression of *ABCC1*, *ABCC2*, *ABCC3*, *ABCC5* transporters in MIA PaCa-2 cells were studied with respect to *PAX2* knockdown. The expression of *ABCC1*, *ABCC2*, *ABCC3*, *ABCC5* transporters were found to be decreased in *PAX2* silenced cells compared to control siRNA transfected cells (Fig 6A).

We wanted to check if PAX2 transcriptional factor can directly bind and regulate the promoters of the transporter genes. To do this, potential PAX2 binding sites were scanned *in silico* within the promoter regions of ABC transporter family of proteins using ConTra v3. Evidence of binding sites were further confirmed from GTRD. With this information, 200bp sequence in the upstream and downstream region of the TFBS were extracted. The potential binding site for PAX2 in the promoter regions of ABC family transporter proteins are given in S9 Fig. Chromatin immunoprecipitation was performed in control siNeg MIA PaCa-2 or siPax2 MIA PaCa-2, using control normal rabbit IgG (Catalogue No#2729, Cell Signalling Technology, USA) or anti-PAX2 rabbit (Catalogue No# ab23799, AbCam Plc, UK) antibody was added (concentration 10μg/ml) and checked for PAX2 promoter occupancy. It was found that PAX2 binds to promoters of *ABCC1*, *ABCC2*, *ABCC3*, *ABCC5* transporters and this binding is decreased upon silencing of *PAX2* in cells. Together these results indicate that PAX2 can be a master regulator of drug resistance in pancreatic cancers by controlling drug transporter cassettes. Inhibitors of PAX2 mediated transcription can be an effective drug target against mixed grade PDAC and can be used to increase susceptibility of end stage PDACs towards existing clinical interventions.

## Conclusions

Many previous reports of drug resistance pancreatic cancers indicated the existence of a molecular network that evolves with the progression of PDACs [76], [77], [78], [79], [80]. We speculate that the poorly differentiated mixed grade nature of PDACs harbor the key to such phenotype [81]. Although various factors like *ABC* family of transporters, regulators like *TM4SF1*, various cofactors had been implicated in drug resistance associated with PDACs [82], [83], [18], [13], [14], [84], none of these were able to distinctly classify them to particular tumor grade. Our analysis observed a distinct pattern variation with respect to histone methylation marks and differential gene expression between low grade and high grade PDAC cell types (Fig 1 and S1 Fig). Further analysis of high grade PDAC cells indicated network of genes that show strong physical correlation with tumor progression in selected pathways (Fig 3 and S5, S6, and S7 Figs). PAX2 served as an oncogenic transcription factor in various other cancers by reprogramming gene regulatory networks [85], [86], [87], [54]. Our data shows that PAX2 binds to the promoter region of ABC family genes and regulate their transcription in high grade PDAC cell lines. In addition, it was also found that PAX2 transcriptional factor can bind to the promoter of previously reported transmembrane-domain family *TM4SF1*, a gene that played a role in gemcitabine resistant pancreatic cancer [83]. Silencing *PAX2* expression resulted in down regulation of *TM4SF1* expression, suggesting a possible mechanism for gemcitabine resistance in PDACs (Fig 6A and 6B). Interestingly, our study indicated that the mechanism of *TM4SF1* driven drug resistance might be transcriptionally controlled by PAX2. These findings indicate that PAX2 could act as a master transcription factor regulating drug resistance phenotype in high grade PDACs.

Surprisingly, drug resistance phenotype is a hallmark of lethality associated with PDACs [88], [89], [90]. We found that *PAX2* silencing alone was able to rescue drug susceptibility in high grade cell types with various chemotherapeutic agents (Fig 5). We checked for survival in PDAC patient samples from various available data sets and performed multivariate analysis on *PAX2* and *ABCC* family members. Surprisingly, we found that *PAX2* in combination with *ABCC* transporters were able to significantly alter hazard ratio and survival in PDAC patient (Fig 6C, 6D, 6E & 6F). Together these findings indicate that *PAX2* may have a major prognostic value and future development of *PAX2* inhibitors warrants clinical interventions.

## Supporting information

S1 Fig Correlation plot and volcano plot for high grade PDAC RNA-Seq data. (A) Correlation plot between RNA-Seq data from DESeq2. (B) Volcano plot of upregulated (red) and downregulated (blue) genes in high grade PDAC cell line.
(TIF)

S2 Fig Activating and repressing function for high grade PDAC cell lines. Activating and repressing function of (A) MIA PaCa-2 H3K4Me1, (B) MIA PaCa-2 H3K4Me3, (C) PANC-1 H3K4Me1 and (D) PANC-1 H3K4Me3. Red lines indicate upregulated genes while the purple line indicates downregulated genes. The black dots represents non-differentially expressed genes (Background). X axis represents the rank of genes based on the regulatory potential while the y axis represents the proportion of genes.
(TIF)

S3 Fig Overlapping genes in high grade PDAC cell lines. Venn diagram for (A) Common upregulated genes between mono and tri-methylated MIA PaCa-2 and PANC-1 and (B) Common downregulated genes between mono and tri-methylated MIA PaCa-2 and PANC-1. (C) Visualization of enriched peaks around the promoter region of *PAX2* gene indicated that trimethylation of histones in that region could possibly lead to transcriptional activity of the underlying gene. This was in agreement with the expression data obtained through RNA-Seq analysis.
(TIF)

S4 Fig Gene Ontology report for biological process, Molecular Function, and Cellular Component of high grade PDAC genes. Gene Ontology report for Biological Process, Molecular Function, and Cellular Component of (A) *DDR2*, (B) *DPYSL5*, (C) *FAM5C*, (D) *PAX2*, and (E) *SPARC* were generated using the FunRich tool. Biological process of *PAX2* presented notable contribution towards negative regulation of apoptosis in general.
(TIF)

S5 Fig Pathview output for the pathway “Pathways in cancer”. Gene components of high grade PDAC were compared against gene components of low grade PDAC, giving rise to the nodes marked in color. Green (-1) depicts genes downregulated in high grade cell line (but upregulated in low grade), while those marked in red (1) depicts upregulated genes in high grade cell line. Some nodes are split between two colors, indicating difference in regulation between MIA PaCa-2 (left) and PANC-1 (right).
(TIF)

S6 Fig Pathview output for the pathway “Transcriptional misregulation in cancer”. Gene components of high grade PDAC were compared against gene components of low grade PDAC, giving rise to the nodes marked in color. Green (-1) depicts genes downregulated in high grade cell line (but upregulated in low grade), while those marked in red (1) depicts upregulated genes in high grade cell line. Some nodes are split between two colors, indicating difference in regulation between MIA PaCa-2 (left) and PANC-1 (right).
(TIF)

S7 Fig Pathview output for the pathway “Pancreatic cancer”. Gene components of high grade PDAC were compared against gene components of low grade PDAC, giving rise to the nodes marked in color. Green (-1) depicts genes downregulated in high grade cell line (but upregulated in low grade), while those marked in red (1) depicts upregulated genes in high grade cell line. Some nodes are split between two colors, indicating difference in regulation between MIA PaCa-2 (left) and PANC-1 (right).
(TIF)

S8 Fig Relative mRNA levels and cell viability assay for high grade PDAC cell lines. (A) MIA PaCa-2 cells were transfected with siRNAs against indicated genes (Gene of Interest, GOI). Relative mRNA levels were analysed with respect to siRNA Negative control (siNEG) 48hours post transfection. Normalization was performed with 18sRNA levels. Experiments were done independently in triplicates SEM = ± 3. (B) Mia-Paca2 cells were transfected using indicated siRNAs for 48hours. Cell viability assay was performed post 48 hours using luminometer. Percent cell viability was calculated with respect to Negative control siRNA (siNeg). siCell Death, Death, an siRNA used as Positive cell death phenotype control, from qiagen, USA was used as a positive control. Experiments were done independently in triplicates SEM = ± 3. (C) PANC-1 cells were transfected with PAX2 siRNAs. Relative mRNA levels were analysed with respect to siRNA Negative control (siNEG) 48hours post transfection. Normalization was performed with 18sRNA levels. Experiments were done independently in triplicates SEM = ± 3.
(TIF)

S9 Fig Predicted binding site for PAX2 in the promoter regions of ABCC family transporter genes. (A) *ABCC1*, (B) *ABCC2*, (C) *ABCC3*, and (D) *ABCC5*.
(TIF)

S1 Table List of upregulated and downregulated genes found in high grade PDAC cell lines using DeSeq2. (XLS)
