# Supplementary figures and images for: An integrated epigenome and transcriptome analysis identifies PAX2 as a master regulator of drug resistance in high grade pancreatic ductal adenocarcinoma
Source: PLoS One. 2019 Oct 17;14(10):e0223554. doi: 10.1371/journal.pone.0223554 (PMC6797122; doi:10.1371/journal.pone.0223554)

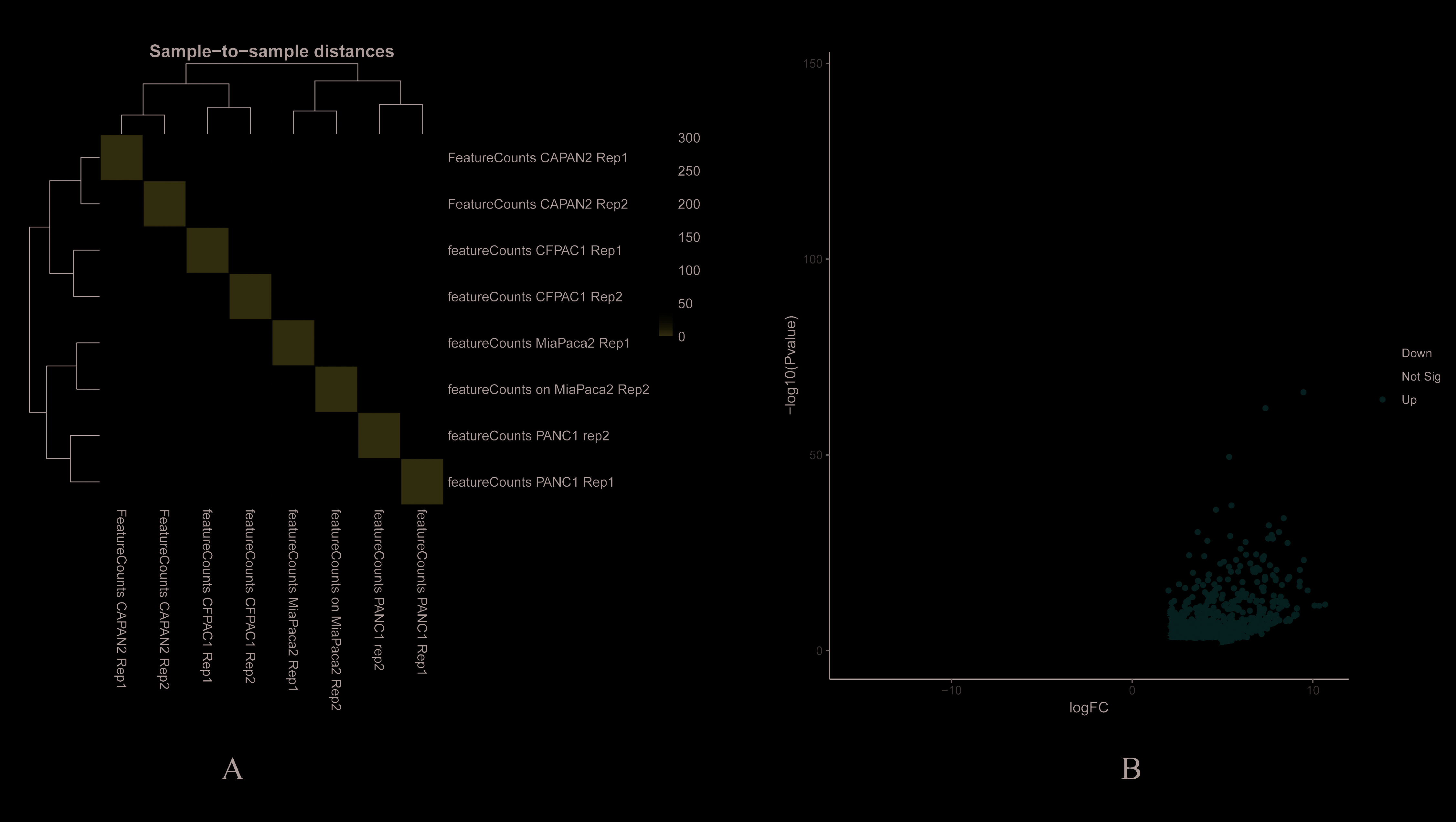

Supplement: S1 Fig — (A) Correlation plot between RNA-Seq data from DESeq2. (B) Volcano plot of upregulated (red) and downregulated (blue) genes in high grade PDAC cell line. (TIF) [file pone.0223554.s001.tif]

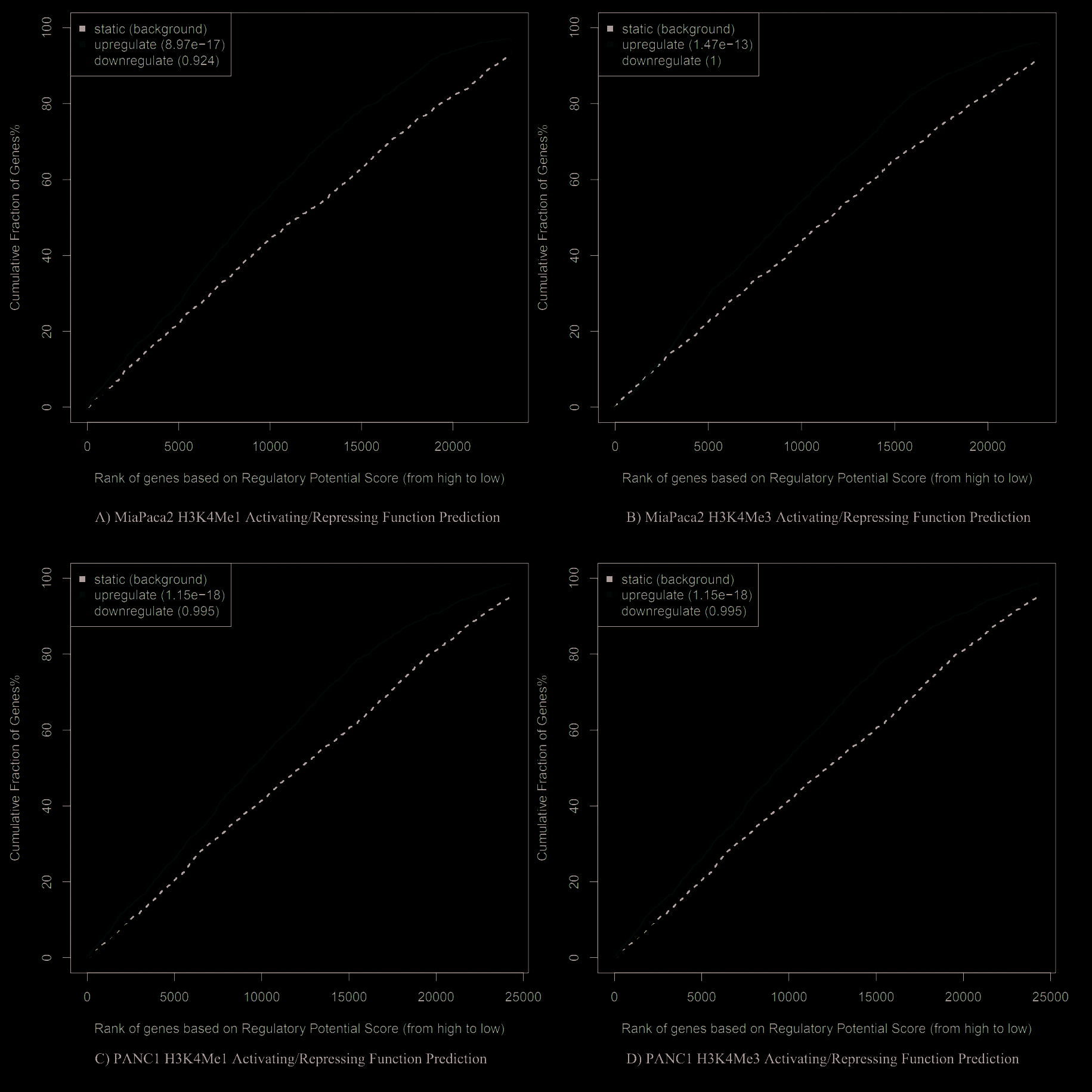

Supplement: S2 Fig — Activating and repressing function of (A) MIA PaCa-2 H3K4Me1, (B) MIA PaCa-2 H3K4Me3, (C) PANC-1 H3K4Me1 and (D) PANC-1 H3K4Me3. Red lines indicate upregulated genes while the purple line indicates downregulated genes. The black dots represents non-differentially expressed genes (Background). X axis represents the rank of genes based on the regulatory potential while the y axis represents the proportion of genes. (TIF) [file pone.0223554.s002.tif]

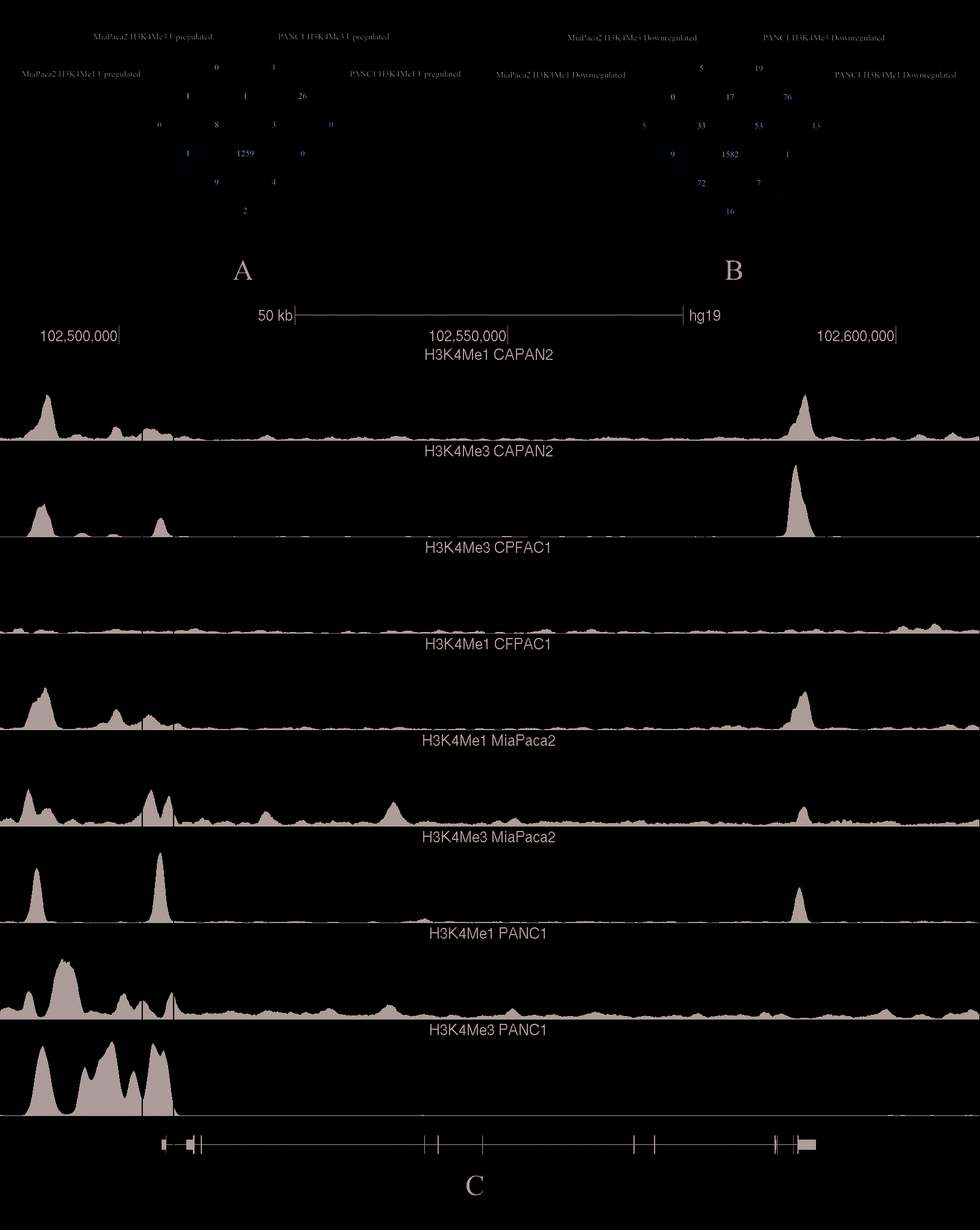

Supplement: S3 Fig — Venn diagram for (A) Common upregulated genes between mono and tri-methylated MIA PaCa-2 and PANC-1 and (B) Common downregulated genes between mono and tri-methylated MIA PaCa-2 and PANC-1. (C) Visualization of enriched peaks around the promoter region of PAX2 gene indicated that trimethylation of histones in that region could possibly lead to transcriptional activity of the underlying gene. This was in agreement with the expression data obtained through RNA-Seq analysis. (TIF) [file pone.0223554.s003.tif]

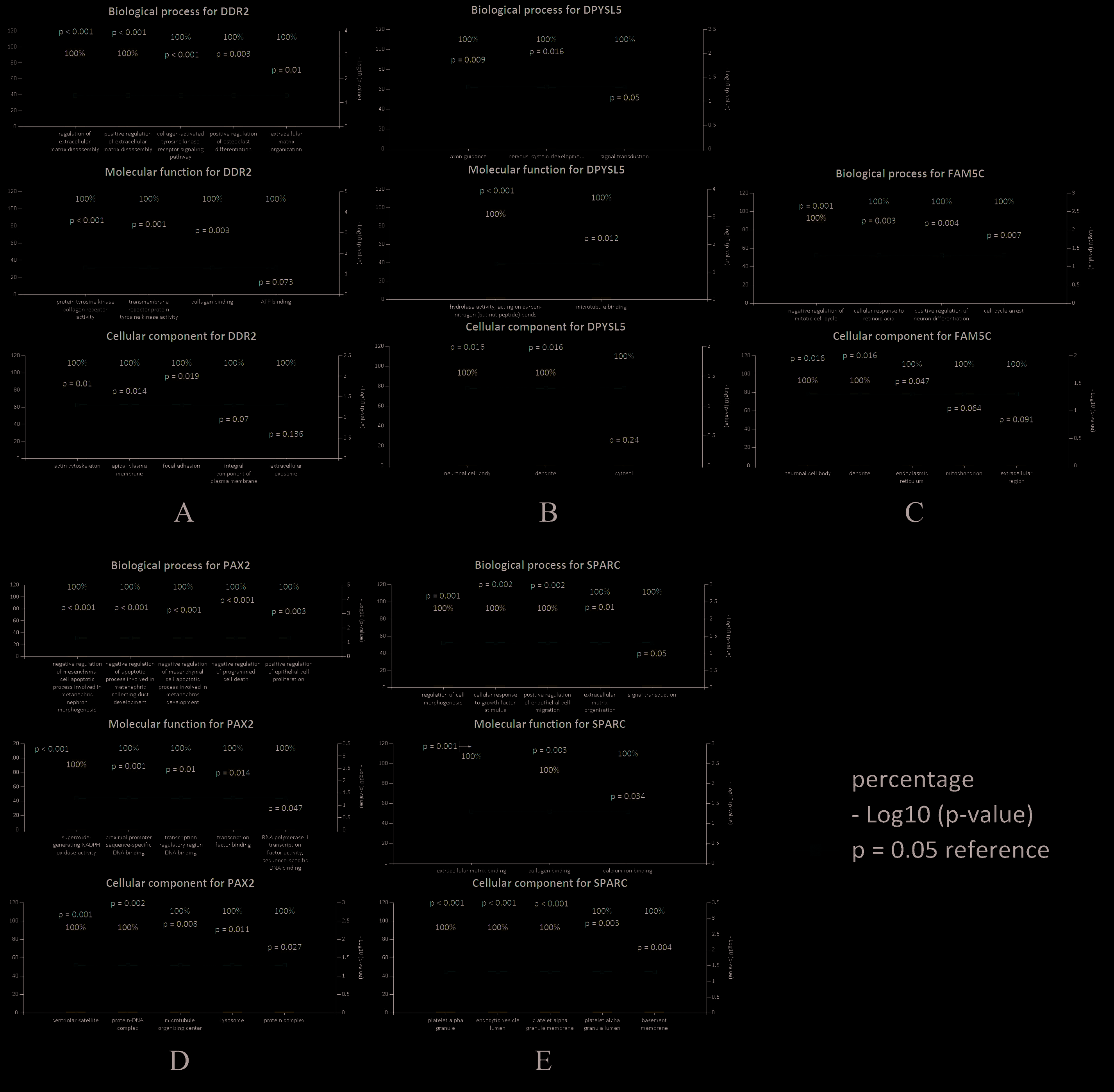

Supplement: S4 Fig — Gene Ontology report for Biological Process, Molecular Function, and Cellular Component of (A) DDR2, (B) DPYSL5, (C) FAM5C, (D) PAX2, and (E) SPARC were generated using the FunRich tool. Biological process of PAX2 presented notable contribution towards negative regulation of apoptosis in general. (TIF) [file pone.0223554.s004.tif]

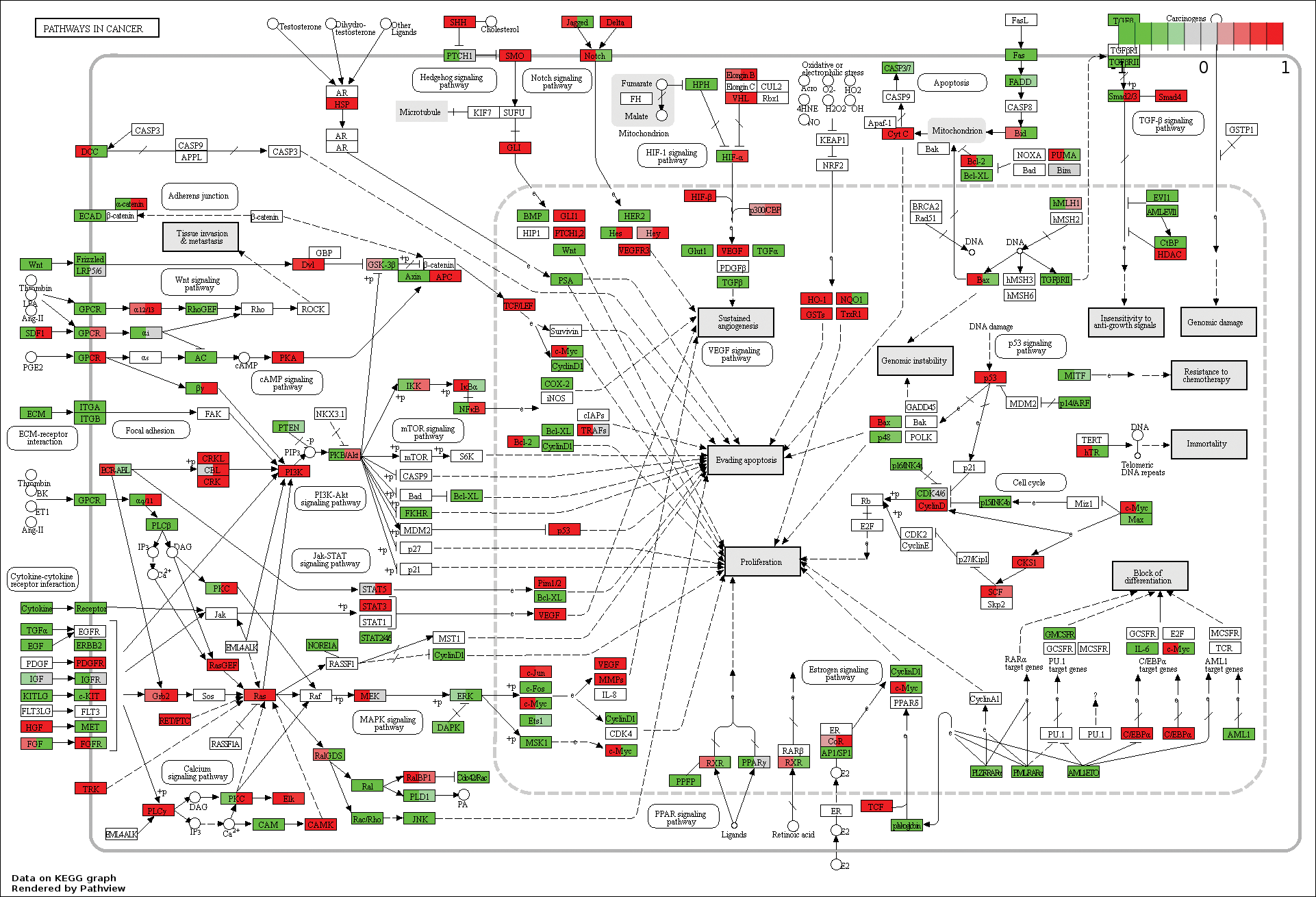

Supplement: S5 Fig — Gene components of high grade PDAC were compared against gene components of low grade PDAC, giving rise to the nodes marked in color. Green (-1) depicts genes downregulated in high grade cell line (but upregulated in low grade), while those marked in red (1) depicts upregulated genes in high grade cell line. Some nodes are split between two colors, indicating difference in regulation between MIA PaCa-2 (left) and PANC-1 (right). (TIF) [file pone.0223554.s005.tif]

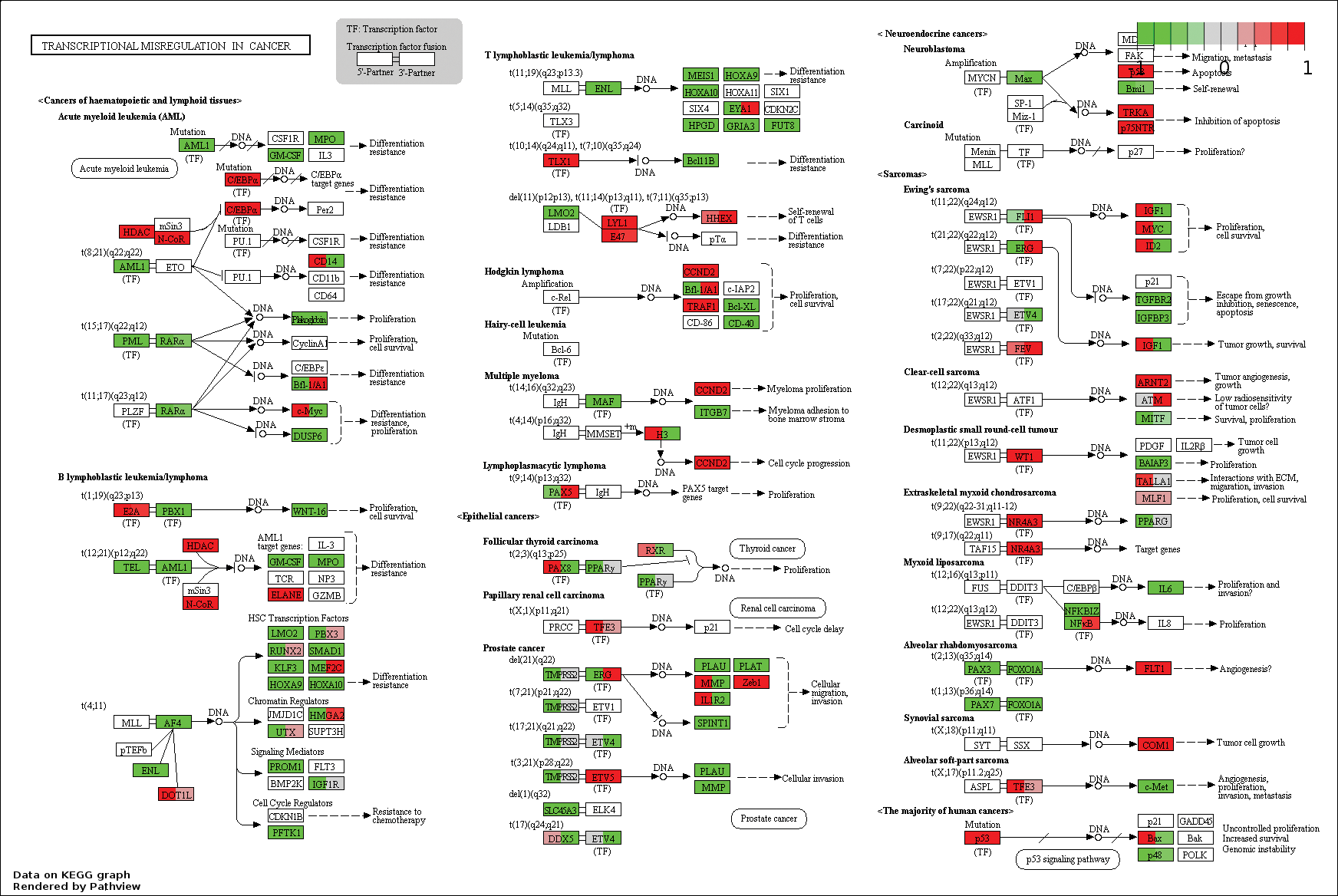

Supplement: S6 Fig — Gene components of high grade PDAC were compared against gene components of low grade PDAC, giving rise to the nodes marked in color. Green (-1) depicts genes downregulated in high grade cell line (but upregulated in low grade), while those marked in red (1) depicts upregulated genes in high grade cell line. Some nodes are split between two colors, indicating difference in regulation between MIA PaCa-2 (left) and PANC-1 (right). (TIF) [file pone.0223554.s006.tif]

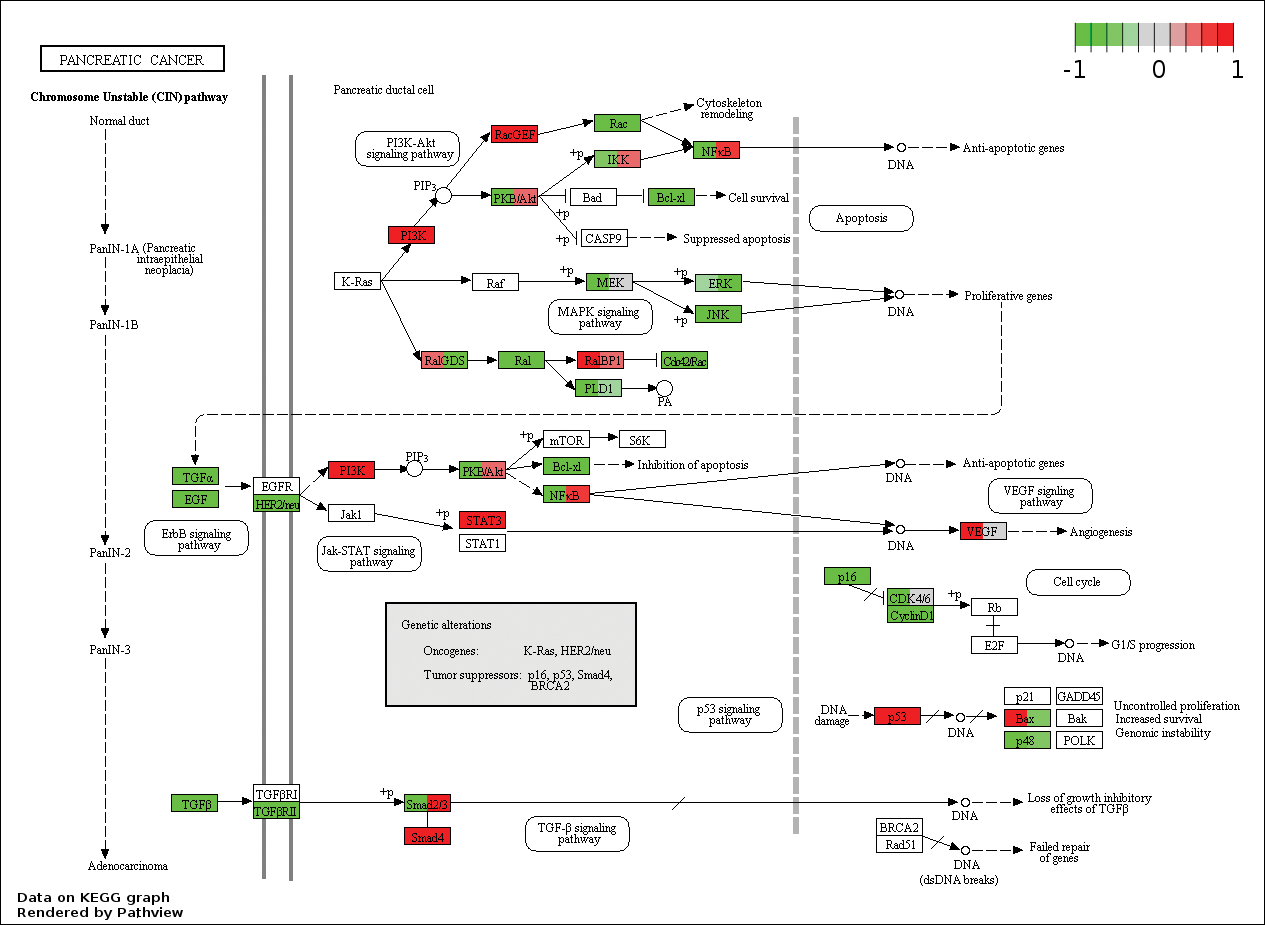

Supplement: S7 Fig — Gene components of high grade PDAC were compared against gene components of low grade PDAC, giving rise to the nodes marked in color. Green (-1) depicts genes downregulated in high grade cell line (but upregulated in low grade), while those marked in red (1) depicts upregulated genes in high grade cell line. Some nodes are split between two colors, indicating difference in regulation between MIA PaCa-2 (left) and PANC-1 (right). (TIF) [file pone.0223554.s007.tif]

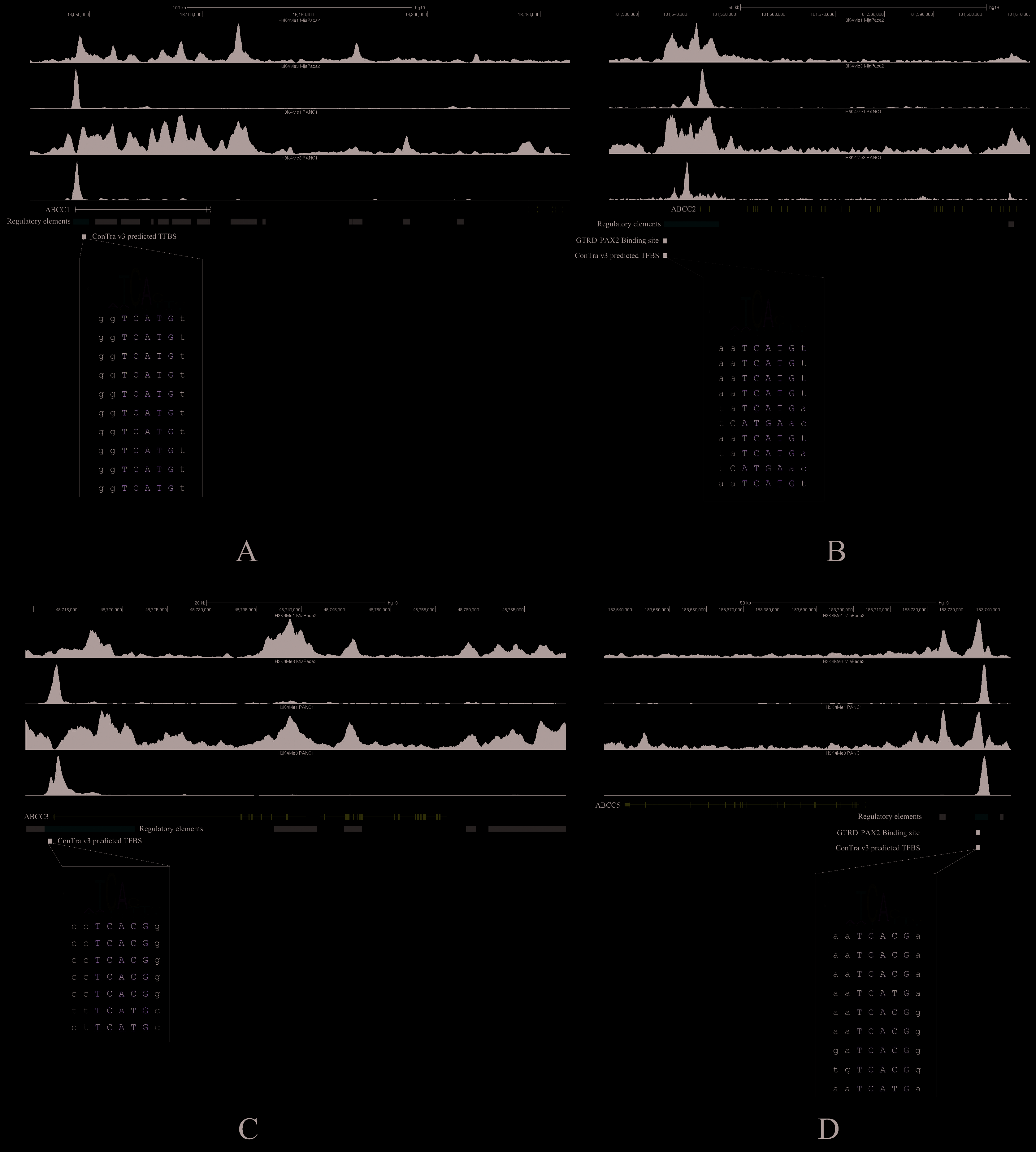

Supplement: S9 Fig — (A) ABCC1, (B) ABCC2, (C) ABCC3, and (D) ABCC5. (TIF) [file pone.0223554.s009.tif]
